# False-Positive IMMY^®^ Serum Cryptococcal Antigen Lateral Flow Assay Results Due to Improper Use of Titration Buffer

**DOI:** 10.3390/jof12020135

**Published:** 2026-02-12

**Authors:** Asmus Tukundane, Wilber Bakka, Mercy Amanige, Dora Babirye, Timothy Mugabi, Abdu Musubire, David B. Meya, David R. Boulware, Brian Doherty, Richard Kwizera, Caleb P. Skipper

**Affiliations:** 1 Infectious Diseases Institute, College of Health Sciences, Makerere University, Kampala P.O. Box 22418, Uganda; bakkawilber@gmail.com (W.B.); amanigemercy@gmail.com (M.A.); dbabirye@idi.co.ug (D.B.); mtimon8@gmail.com (T.M.); amusubire@yahoo.com (A.M.); david.meya@gmail.com (D.B.M.); kwizerarichard@ymail.com (R.K.); 2 Faculty of Pharmaceutical Sciences, James Lind Institute, Rue de la Cité 1, 1204 Geneva, Switzerland; 3 Division of Infectious Diseases and International Medicine, Department of Medicine, University of Minnesota, Minneapolis, MN 55455, USA; skipp015@umn.edu (C.P.S.); 4 IMMY Diagnostics, 2701 Corporate Centre Drive, Norman, OK 73069, USA; brian-doherty@immy.com

**Keywords:** cryptococcal antigen, lateral flow assay, false positive, specimen diluent, titration diluent, heterophilic antibodies

## Abstract

The IMMY cryptococcal antigen (CrAg) lateral flow assay (LFA) is a reliable diagnostic tool for *Cryptococcus* detection, but false-positive results may arise from procedural or reagent-related errors, underscoring careful operation of the assay to ensure diagnostic accuracy and prevent unnecessary treatment. Two patients who were initially reported as CrAg-positive by a peripheral laboratory were referred to Kiruddu Hospital in Kampala, Uganda, for clinical assessment and confirmatory testing. Repeat tests were conducted using specimen diluent following the manufacturer’s protocol, resulting in negative results. Semi-quantitative CrAg LFA testing and a series of control assays were performed to identify the source of error. We were able to consistently reproduce positive results when the titration diluent was inappropriately used instead of the specimen diluent. Serial dilutions confirmed persistent false positivity up to 1:80 when inappropriately using the titration diluent, while all dilutions that appropriately started with the specimen diluent remained negative. We hypothesize that the incorrect use of titration diluent instead of specimen diluent led to false-positive CrAg LFA results due to the absence of the blocking agent that neutralizes heterophilic antibodies. Procedural errors can lead to diagnostic misinterpretation and serious consequences in clinical management, emphasizing the importance of adherence to manufacturer’s instructions.

## 1. Introduction

Cryptococcosis is an opportunistic fungal infection that mainly affects individuals with compromised immune systems [1]. In persons with advanced HIV disease, cryptococcal meningitis accounts for approximately 19% of all AIDS-related deaths globally [2]. Rapid diagnosis is critical for improving treatment outcomes. The introduction of the cryptococcal antigen lateral flow assay (CrAg LFA; IMMY, Norman, OK, USA) has greatly improved the early detection of cryptococcal disease. This assay offers several advantages, including a short turnaround time of less than 10 min, no requirement for specimen pretreatment, ease of use without the need for specialized laboratory infrastructure, and affordability [3]. The test has a sensitivity for serum CrAg of 99.3% and specificity of 99.1% among persons with HIV [4]. However, diagnostic inaccuracies with the CrAg LFA, though uncommon, may arise from several sources, including user errors, the prozone effect at very high antigen concentrations, antigen cross-reactivity with other organisms, and interference from heterophilic antibodies and autoantibodies such as rheumatoid factor [5]. Importantly, inadequately trained personnel, inconsistent adherence to standardized procedures, and improper reagent handling or substitution remain preventable contributors to compromised test accuracy [6].

We present a real-life scenario where improper test utilization due to procedural error resulted in false-positive test results. This scenario is a clear demonstration of how improper reagent use, specifically substituting titration diluent for specimen diluent, can cause false-positive CrAg LFA results. Such procedural errors can lead to patient mismanagement and unnecessary antifungal therapy for 6–12 months.

## 2. Clinical Scenario and Case Description

### 2.1. Clinical Scenario

This was a laboratory-based observational investigation involving two patients with HIV referred to the Infectious Diseases Institute (IDI) Meningitis Study Team at Kiruddu Hospital, Kampala, Uganda. Both patients had been reported as serum CrAg-positive by a peripheral laboratory and were referred within the same week to rule out cryptococcal meningitis. Upon receipt by the study team, repeat point-of-care CrAg LFA testing was conducted according to manufacturer’s instructions, and both samples resulted in negative results. Additionally, clinical assessment of both patients by the study team found no symptomatic evidence for disseminated cryptococcosis or cryptococcal meningitis. One of the two patients gave consent for including their clinical history as follows.

### 2.2. Case Description

The consented patient (referred to as Patient A) was a 62-year-old female with a history HIV diagnosis approximately 15 years ago. She was ART-experienced with an outside report of good adherence. Her CD4 count at the time of presentation was 264 cells/μL. She had a past medical history significant for prior cerebrovascular accidents (CVAs) with left-sided weakness in the prior 7 years but with minimal residual deficits. Her only medication besides ART was amlodipine for hypertension. She had no history of heart disease or autoimmune disease.

Patient A presented to our study site after a sudden collapse followed by right-sided weakness and difficulty in speaking, with symptoms noted for one day at an outside hospital. There was no reported history of headache, dizziness or vomiting. Her general exam showed she was of good nutritional status, not anemic, had no jaundice, and no edema of the limbs. She was noted be afebrile at presentation (36.5 °C), with normal respiratory rate of 16 breaths per minute and oxygen saturation of 98% on room air, but with bradycardia at a heart rate of 47 beats per minute. Blood pressure was recorded at 124/76. Her Glasgow Coma Scale score at initial assessment was 7 of 15 (E = 1, V = 1, M = 5). Nuchal rigidity was not noted, and Kernig’s sign was negative. Abdominal exam and chest examinations were normal. Her motor examination revealed predominant right-sided weakness: right upper limb 2/5, right lower limb 3/5, left upper limb 4+/5, and left lower limb 4+/5 with normal tone and reflexes. Sensation to pain was present.

The initial assessment was concerning for recurrent CVA, with a possible associated seizure. However, due to the immunocompromised status and persistent encephalopathy, central nervous system infection was considered in the differential. In addition, the patient arrived with documents from the outside facility indicating a positive serum CrAg. Thus, additional infectious disease investigations were ordered, including repeat CrAg testing.

Investigations done at admission revealed that random blood glucose was 6.4 mmol/L. Complete blood count demonstrated white blood cell levels of 4900 cells/μL, hemoglobin 12.7 g/dL, and platelets of 469,000 cells/μL. Random blood glucose was 4.6 mmol/L. Renal function was normal (creatinine 0.72 mg/dL) with unremarkable electrolytes. Liver function laboratories (total bilirubin = 0.373 mg/dL, ALP = 88 U/L, ALT = 39 U/L) were also found normal. ECG demonstrated sinus bradycardia. Transthoracic echocardiography revealed a left ventricle ejection fraction of 58%, along with mild mitral valve regurgitation. Chest X-ray was unremarkable. The brain CT scan revealed an old right middle cerebral artery infarct and a new left middle cerebral artery infarct of medium size, with minimal cerebral edema. Blood cultures collected at presentation showed no growth, including no growth of *Cryptococcus* yeast. Urine TB lipoarabinomannan (LAM) was negative. Repeat serum CrAg testing by the hospital team was also negative.

The patient was treated for recurrent ischemic stroke with aspirin and atorvastatin and continued amlodipine. During hospitalization, the patient developed an episode of aspiration pneumonia that was treated with ceftriaxone with improvement. Over the next few days, the patient’s GCS improved to 11/15 (E = 4, V = 2, M = 5), and she was discharged with speech difficulty and right-sided hemiparesis.

## 3. Methods and Results

To assess whether the initial laboratory results represented false positives, we conducted a series of experiments on the same serum samples under controlled laboratory conditions. The testing procedures described below outline our methodology, the subsequent results, and the rationale for concluding that the initial results were false positives, most likely caused by deviations from the manufacturer’s instructions. All test kits employed in this study were verified to be within their expiration dates and appropriately stored prior to use.

### 3.1. CrAg LFA Qualitative Testing and Titer Procedure

**Figure 1:**
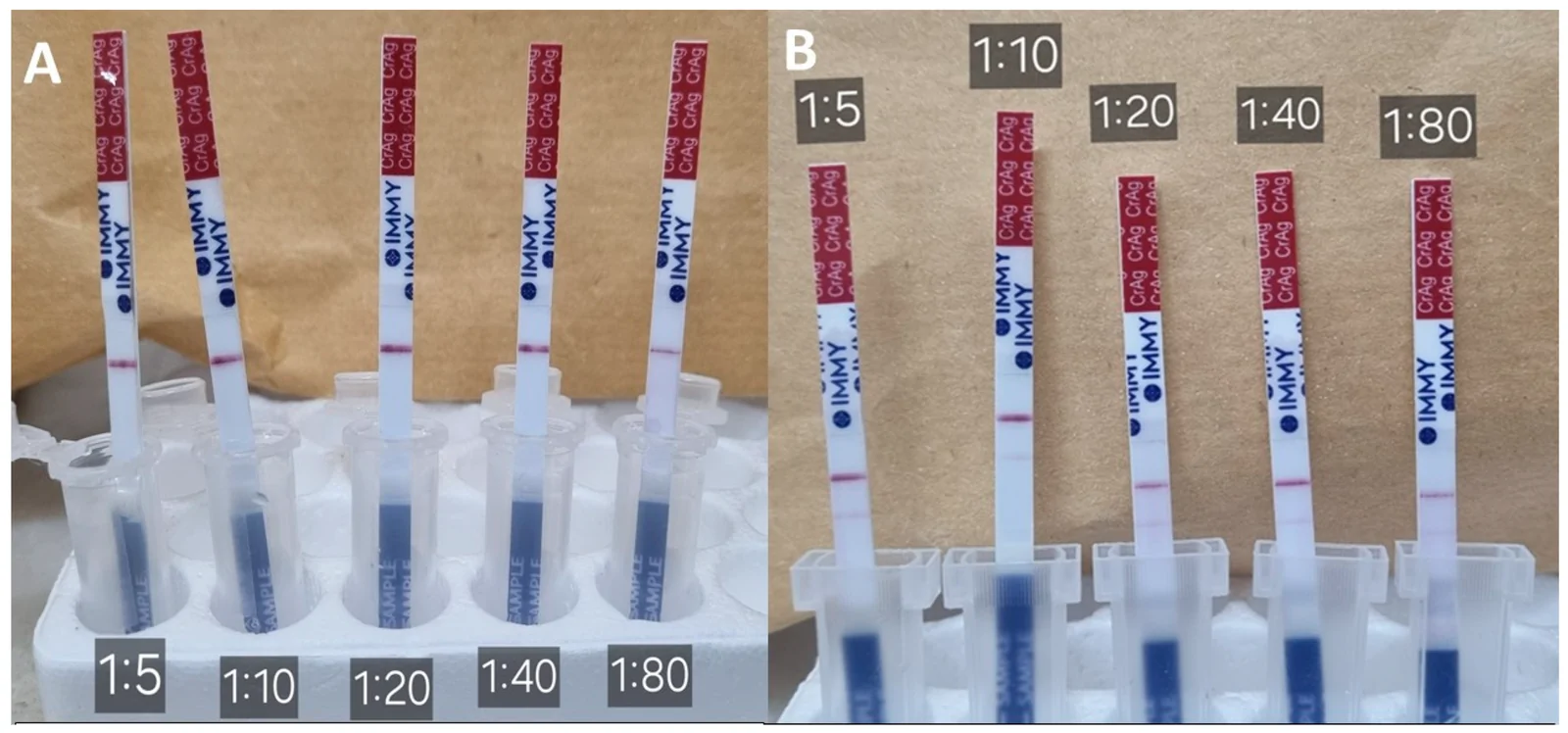
Cryptococcal antigen lateral flow assay with correct and incorrect diluents (Sample A pictured). Subpanel (**A**) shows the use of correct specimen diluent which yielded true-negative results. Serial dilutions using the specimen diluent as per manufacturer’s instructions, resulted in the IMMY CrAg LFA remaining a true negative for the two samples. Subpanel (**B**) shows the use of incorrect titration diluent which yielded false-positive results. Substituting specimen buffer with titration diluent at the 1:5 dilution step resulted in false-positive bands of consistent intensity up to the 1:80 dilution for both samples.

CrAg testing was performed on both patients’ serum (hereafter labeled Sample A and Sample B) following IMMY manufacturer guidelines. We first performed qualitative testing with the CrAg LFA using the appropriate ratio of serum sample to specimen diluent. Samples A and B both tested negative. We next performed the same qualitative testing replacing the specimen diluent with the titration diluent and noted that Samples A and B both tested positive. We then conducted serial dilutions using the CrAg LFA to determine CrAg titers and verify the role of the diluent in producing accurate results. First, mixing 40 µL of serum with 160 µL of specimen diluent (1:5 dilution) yielded negative results for Samples A and B. Subsequent serial dilutions of 1:10, 1:20, 1:40, and 1:80 remained negative when using the titration diluent as per manufacturer’s instructions (Figure 1A). We then substituted the specimen diluent with the titration diluent at the initial 1:5 dilution step. Doing so resulted in false-positive bands of consistent intensity starting at the 1:5 titer and continuing through the 1:80 titer dilution (Figure 1B).

### 3.2. IMMY CrAgSQ Procedure

We also performed confirmatory testing using the IMMY CrAgSQ assay, following the manufacturer’s instructions. Samples A and B tested negative on the CrAgSQ platform. The CrAgSQ assay has been shown to demonstrate excellent diagnostic performance, maintaining the high sensitivity and specificity of the standard CrAg LFA while providing semi-quantitative grading of antigen levels, but mitigating the erroneous results that may occur with extreme antigen concentrations (i.e., prozone effect) [7,8].

### 3.3. Negative Controls and Lot Numbers

To ensure that diluent contamination was not the cause of the false-positive results, we ran negative controls. We set up a three-condition experiment without including any serum sample: adding 80 μL of specimen diluent to tube #1, adding 80 μL of titration diluent to tube #2, and adding 40 μL of specimen diluent and 40 μL of titration diluent to tube #3. All three negative control conditions resulted in negative test results.

Lastly, we took a random, unrelated serum sample left over from routine HIV screening and ran CrAg LFA using the appropriate specimen diluent, which tested negative. We then ran that same unrelated serum sample, intentionally replacing the specimen diluent with the titration diluent. This also led to a negative result.

To ensure we were not encountering a phenomenon related to error or variation in specific lot manufacturing, we repeated the qualitative LFA experiments with Samples A and B and the negative control experiments, using test strips and reagents from a different manufacturing lot number. All results remained consistent as previously described.

## 4. Discussion

Our investigation identified the specific conditions under which the observed false-positive CrAg LFA results occurred, which we strongly suspect was caused by heterophile antibody interference. These conditions involved a combination of serum from particular patients and the use of the titration diluent in place of the specimen diluent. The negative control experiments allowed us to rule out diluent contamination. An audit of the referring laboratory indeed revealed that titration diluent had been used instead of specimen diluent to run the CrAg LFA tests.

**Figure 2:**
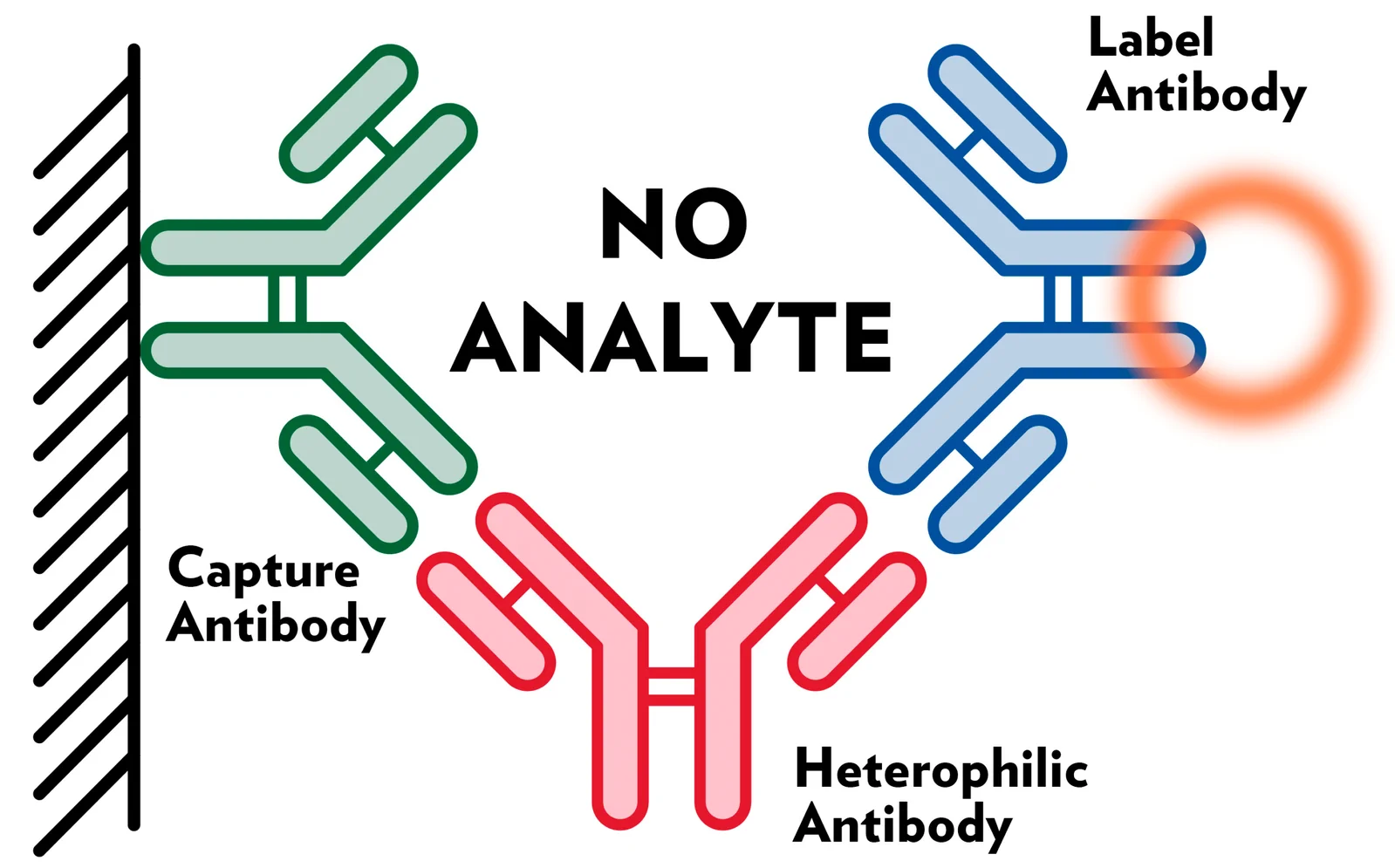
Suspected mechanism of false positives and the role of heterophilic antibody. Typically, a lateral flow sandwich immunoassay requires the target antigen to conjugate the capture antibody and the label antibody. However, when heterophile antibodies are present, their non-specific binding can inappropriately link the capture antibody with the label antibody, resulting in a colorimetric change and line to form on the test strip, resulting in a false-positive reading.

This report confirms that the improper use of titration diluent can cause false-positive CrAg LFA results under specific circumstances. The specimen diluent contains a blocking agent that neutralizes heterophilic antibodies, such as human anti-mouse antibodies (HAMAs) and rheumatoid factor (RF), thereby preventing non-specific binding [9]. In the absence of such blocking buffer or agent, heterophilic antibodies in the patient’s sample can non-specifically link the capture and detection antibodies, forming a false immune complex that produces a signal even in the absence of the target analyte, leading to a false-positive result, as shown in Figure 2.

Heterophile antibodies are naturally occurring antibodies that are characterized by polyspecificity and typically exhibit weak binding affinity for targets. While heterophile antibodies can occur through homeostatic V(D)J somatic gene recombination, certain triggers can increase their prevalence; classic examples include Epstein–Barr virus infection and autoimmune diseases such as systemic lupus erythematosus [10]. Anti-animal antibodies, such as human anti-mouse antibodies, are also a primary source of heterophile antibodies [11]. Although assay manufacturers routinely include blocking agents in their formulations, not all heterophilic interferences can be neutralized by the blocking immune globulins, even when pooled from multiple species, as some heterophilic antibodies may react with idiotypes absent from the blocking agent [12]. To mitigate this risk, each IMMY production lot is validated to effectively block at least 40 µg/mL of human anti-mouse antibodies, ensuring improved assay specificity and reliability.

The titration diluent in the IMMY CrAg LFA kit lacks this blocking component and is formulated for use in serial dilutions beginning at 1:10 and above during semi-quantitative testing. Direct use of titration diluent without first applying specimen diluent to serum would permit non-specific heterophile antibody binding—potentially leading to false-positive test results. However, as not all human serum samples will include heterophile antibodies capable of causing non-specific binding, using the titration diluent without the specimen diluent may also result in accurate test results. This intrinsic observation by laboratory staff may lead to a “laissez-faire” approach to ensuring that the correct diluent is used. LMIC settings may be particularly susceptible to these errors as reagents may be in short supply, and there is limited testing capacity (and thus limited awareness) for heterophilic antibodies such as HAMAs or RF. Overall, these observations underscore the need for proper reagent management, laboratory competency training, and adherence to standard operating procedures; and align with the current manufacturer’s instructions.

It is important to note that heterophile antibodies affect antibody-based diagnostic assays beyond lateral flow assays. The cryptococcal latex agglutination assay requires a protease treatment to alleviate any heterophilic antibody binding, while ELISA technologies typically utilize blocking reagent to minimize the effect of heterophilic antibodies. Further, erroneous results occur beyond heterophile antibody interference. Our study team have seen false-positive or negative results attributable to using incorrect sample-to-diluent volumes, reading strips before the 10 min result time, and mistakenly using the positive control as specimen diluent.

Addressing these gaps through strengthened laboratory quality systems, regular training, and adherence to manufacturer guidelines is key to improving diagnostic accuracy and patient outcomes. In laboratories that do not perform CrAg titers, as well as those conducting only point-of-care (POC) testing for patient care and management in places such as in hospital wards and emergency centers, discarding the titration diluent after running a positive control could be considered an operational step when opening a new CrAg test kit.

Enhanced product design could greatly support laboratory staff and healthcare professionals by incorporating color-coded labels, varied bottle sizes, distinct cap shapes, and differentiated buffer packaging. Including clear procedural guidance on the appropriate use of each reagent at specific stages of testing would further enhance accuracy. These visual and procedural distinctions would help prevent mix-ups between diluents, reduce handling errors, and promote greater precision and reliability in routine laboratory operations.

### Study Limitations

This small technical note based on limited observational data cannot estimate the prevalence of the heterophile antibody interference in our population, and generalizability is limited. Further, we were not able to perform specific investigations into our individual patient samples for the presence of common heterophilic antibodies with our current laboratory capacity in Uganda. As this started as an active investigation to ensure proper clinical care, none of the technicians were blinded through the series of experiments. Despite these limitations, we believe our technical observation is an important reminder on the mechanisms by which false-positive (and negative) results may occur even in highly sensitive and specific immunoassays.

## 5. Conclusions

Accurate CrAg LFA testing depends on the correct use of the included assay buffers. Titration diluent should be reserved strictly for serial dilutions (1:10 and above), while specimen diluent must be used for all qualitative testing and the initial 1:5 titration step. Regular staff training, reagent inventory control, and strict compliance with manufacturer protocols are essential to prevent diagnostic errors and ensure reliable patient outcomes. The report highlights a critical procedural error that can result in diagnostic misinterpretation and lead to serious consequences in clinical management, emphasizing the need for strict adherence to manufacturer’s instructions and internal quality assurance measures in diagnostic laboratories.

## Data Availability

All data generated or analyzed during this study are included in this published article. The authors confirm that all data underlying the findings are fully available without restriction and can be availed by contacting Mr. Asmus Tukundane (asmus.tukundane@gmail.com).

## Abbreviations

The following abbreviations are used in this manuscript:

AIDS	Acquired Immunodeficiency syndrome
CrAg	Cryptococcal antigen
LFA	Lateral flow assay
IDI	Infectious Diseases Institute
SQ	Semi quantitative
LMIC	Low- and middle-income country
RF	Rheumatoid factor
POC	Point of care
